# Inhibitory Control, but Not Prolonged Object-Related Experience Appears to Affect Physical Problem-Solving Performance of Pet Dogs

**DOI:** 10.1371/journal.pone.0147753

**Published:** 2016-02-10

**Authors:** Corsin A. Müller, Stefanie Riemer, Zsófia Virányi, Ludwig Huber, Friederike Range

**Affiliations:** 1 Messerli Research Institute, University of Veterinary Medicine Vienna, Medical University of Vienna and University of Vienna, Vienna, Austria; 2 Department of Cognitive Biology, University of Vienna, Vienna, Austria; 3 Animal Behaviour, Cognition and Welfare Research Group, School of Life Sciences, University of Lincoln, Lincoln, United Kingdom; CNR, ITALY

## Abstract

Human infants develop an understanding of their physical environment through playful interactions with objects. Similar processes may influence also the performance of non-human animals in physical problem-solving tasks, but to date there is little empirical data to evaluate this hypothesis. In addition or alternatively to prior experiences, inhibitory control has been suggested as a factor underlying the considerable individual differences in performance reported for many species. Here we report a study in which we manipulated the extent of object-related experience for a cohort of dogs (*Canis familiaris*) of the breed Border Collie over a period of 18 months, and assessed their level of inhibitory control, prior to testing them in a series of four physical problem-solving tasks. We found no evidence that differences in object-related experience explain variability in performance in these tasks. It thus appears that dogs do not transfer knowledge about physical rules from one physical problem-solving task to another, but rather approach each task as a novel problem. Our results, however, suggest that individual performance in these tasks is influenced in a complex way by the subject’s level of inhibitory control. Depending on the task, inhibitory control had a positive or a negative effect on performance and different aspects of inhibitory control turned out to be the best predictors of individual performance in the different tasks. Therefore, studying the interplay between inhibitory control and problem-solving performance will make an important contribution to our understanding of individual and species differences in physical problem-solving performance.

## Introduction

Human infants naturally develop an understanding of the physical world through observation of regularities which extend and/or confirm innate predispositions [1–3]. Playful exploration, including manipulating objects and bringing them in relation to each other, is an important basis for this development and allows detection of the objects’ affordances [4,5]. During such exploration, infants spontaneously search for causal explanations for their observations and engage in “hypothesis testing” activities to confirm their expectations [5–9]. In addition, exploration involves practicing motor sequences that can serve as precursors to later problem-solving behaviour [4].

Also for animals it has been suggested that playful object exploration may lead to affordance learning and honing of mechanical skills [10–12]. Some support for this suggestion is provided by a comparative analysis that linked object play to extractive foraging and tool use in primates [13]. In addition a causality bias during associative learning has recently been described also for chimpanzees (*Pan troglodytes*): they learn about causal cues more quickly than about arbitrary cues [14]. Opportunities to manipulate objects, to learn about their affordances and to detect causal relationships may therefore have substantial influences also on animals’ cognitive skills in the physical domain.

A wealth of data has accumulated in recent years that documents substantial differences between species in their physical problem-solving abilities, which is typically explained by differing selection pressures exerted by the ecology and environment of the species together with morphological and phylogenetic constraints [15–20]. In addition, many studies have described sometimes vast individual differences in performance in cognitive tasks also within species (e.g. [21–24]). Exploring the basis of these individual differences is of prime relevance for our understanding of animal intelligence [23].

As learning plays an important role for problem-solving performance, it appears likely that differences in prior experience are the basis of at least some of the observed individual differences in problem-solving performance. Consequently, it is increasingly becoming standard practice to report prior participation in similar experiments for subjects tested in problem-solving tasks (e.g. [20,25–27]). In addition, where the opportunity arose, researchers investigating cognitive abilities of animals in the physical domain have started testing whether subjects with more experience in similar tasks, typically in terms of participation in earlier experiments, outperform more naïve subjects (e.g. [28–31]). These analyses showed for example that chimpanzees with prior experience with a two-trap box outperformed their less experienced conspecifics in two other trap tasks [30], or that great apes with pre-experience with an obstacle task performed better in a trap-platform task and a barrier-platform task than subjects without the same experience [31]. In addition, Cunningham and colleagues found that prior exposure to the rake led to improved performance in a subsequent raking task in female (but not in male) gibbons (Hylobatidae) [32]. Similarly, an early study by Thompson and Heron [33] found that Scottish terriers raised under impoverished conditions performed poorly in barrier and maze tests compared to terriers raised as normal pets. In contrast to these studies, Brent found no effect of prior experience with reaching tools on the performance of chimpanzees in a tool-reaching task [34], and Osthaus and colleagues found no effect of prior training with two short parallel strings on the subsequent performance of dogs with other string-pulling arrays [29].

As outlined, the extent to which physical problem-solving abilities are influenced by (or dependent on) prior experiences currently still remains unclear. This is at least partly due to the fact that many of the existing analyses stem from studies that were not specifically designed to address the question of the influence of prior experiences on individual problem-solving performance. Instead they describe post-hoc analyses of experiments, in which limitations in availability necessitated the use of subjects with different experimental histories. In addition, most of the existing studies investigating effects of prior experience are short-term (i.e., testing for transfer between tasks within studies or between consecutive studies; e.g. [28–32,35]). However, subjects accumulate experiences not only in the short-term but over their lifetime. In addition, opportunities to learn about objects’ affordances already in early life may be particularly relevant for the development of an understanding of the physical world. Indeed, differences in rearing conditions, in particular in terms of access to diverse manipulanda, have been suggested as a likely explanation for striking population differences in physical problem-solving skills, for example between different captive populations of chimpanzees (e.g. [36,37]), or between captive and wild kea (*Nestor notabilis*) [17]. To evaluate the extent to which experiences with object manipulation influences future physical problem-solving skills, it is necessary to compare groups of subjects that differ in their experiences accumulated over the lifetime, with type and amount of experience manipulated by the experimenters and random assignment of subjects to the different groups. This approach was therefore implemented in the present study.

In addition or alternatively to prior experiences, also other individual-specific factors may influence a subject’s problem-solving performance. In particular the subject’s level of inhibitory control is assumed to have consistent effects on problem-solving performance: subjects with better inhibitory control should perform better in problem-solving tasks than those with poor inhibitory control, who will be easily distracted by irrelevant but salient cues and/or will fail to take into account the complete setup of the task as their attention is captured by a single salient stimulus (such as a food reward) [38]. In humans, the level of inhibitory control has been linked to a variety of cognitive performance measures in children and adolescents [39–43], and a recent comparative analysis suggested a robust evolutionary relationship between the level of inhibitory control and brain size across a wide range of animal taxa [44]. Therefore, we expected that an individual’s performance in physical problem-solving tasks would also be influenced by its level of inhibitory control at the time of testing. This association could appear in at least two ways: poor inhibitory control may interfere with an individual’s ability to apply its knowledge to the problem at hand or with its ability to learn to solve the task.

Domestic dogs are a suitable species to investigate factors affecting individual problem-solving performance. They are available in sufficient numbers and they are highly amenable to experimental studies, which made them one of the most popular species for studies of animal cognition in recent years [45,46]. While the main focus of dog cognition research has been on their skills in the social domain, which may exceed even those of great apes in some tasks [47–49], an increasing number of studies have also investigated the performance of dogs in physical problem-solving tasks. The first studies suggested that the abilities of dogs in the domain of physical cognition are poor compared to other mammals due to their history of domestication, which may have led to alleviated selection pressures in this domain [50,51]. Recent evidence does not support this hypothesis, however, as wolves did not perform better than dogs in string-pulling tasks [52] or object permanence tasks [53] (see also [54] for similar evidence in domestic pig and wild boar). Instead, another explanation may account for the comparably poor performance of modern domestic dogs in many physical problem-solving tasks: limited opportunities to gain experiences in this domain. Compared to primates (including humans), young pet dogs typically have fewer opportunities to learn about means-end connections and other contingencies that may facilitate problem-solving performance in the physical domain (indeed, many dog owners and dog trainers consider it undesirable if a dog manipulates objects such as doors, drawers etc.). If experience plays a relevant role for the problem-solving performance in dogs, we therefore predict that offering them ample opportunities to learn about physical contingencies in their early lives will lead to an improved performance in physical problem-solving tasks later-on. As the baseline level of physical cognition skills in dogs is rather low compared to other species, the potential for improvement is high. In addition, as dogs reach adulthood in less than two years, studies manipulating their experience over prolonged periods with subsequent testing as (young) adults are feasible.

Here, we report the results of a 3-year study, in which dogs were assigned to one of three different experience groups as puppies. For all these dogs, the level of inhibitory control was assessed with three impulse control tasks and they were tested in four physical problem-solving tasks as young adults. Two groups of puppies were supplied with a series of manipulative toys between the age of 3 and 15 months. One (the enriched group) was supplied with a set of toys that offered the dogs opportunities to learn about means-end connections, effects of gravity and other contingencies involved in the physical problem-solving tasks presented to them as adults. In order to differentiate between effects of specific relevant experiences and effects of purely manipulative experiences (performing certain actions/movements), the second group of puppies (the manipulative group) was supplied with a set of similar but perceptually opaque toys that did not offer the same learning opportunities. The third group of puppies (the control group) was not supplied with extra toys but grew up with only balls, ropes, rubber toys and similar objects for stimulation, as is customary for pet dogs in Austria. We predicted that the extensive experiences of the subjects in the enriched group would result in an improved performance in the physical problem-solving tasks compared to the control group. If the dogs in the enriched group learned about relevant contingencies, we predicted that they would outperform also the subjects in the manipulative group. If, in contrast, the extensive manipulative experience led to an improved performance in the physical problem-solving tasks, we predicted no difference between the two experimental groups and that the subjects in both experimental groups would outperform the subjects in the control group. In addition, we predicted that dogs with better inhibitory control scores would perform better in the physical problem-solving tasks, as better inhibitory control would give them a better chance to perceive relevant perceptual information before making a decision.

## Methods

### Ethics statement

All procedures were performed in compliance with the Austrian Federal Act on the Protection of Animals (Animal Protection Act–TSchG, BGBl. I Nr.118/2004). All of the reported experiments were completely non-invasive and therefore, according to the Austrian Animal Experiments Act (§ 2, Federal Law Gazette No. 501/1989), are not considered as animal experiments and do not require obtaining special permission. The person recognizable on three of the Supporting Information video clips (S4, S5 and S6 Movies) has given written informed consent (as outlined in the PLOS consent form) to publish these video clips.

### Subjects

All subjects lived as pet dogs with their owners, who volunteered to participate in this study and provided written consent. The study was restricted to a single breed to exclude possible variations due to breed differences. Sixty-three young Border Collies were recruited. Of these, 22 dogs dropped out at some point during this long-term study, mostly due to time limitations of their owners. The remaining 41 dogs (16 males and 25 females) completed the study (though not all subjects completed all tests, cf. Table 1).

**Table 1:** Number of subjects in the respective tasks.

Task	Number of subjects (males/females)
*Experience treatments*	
Enriched group	14 (4/10)
Manipulative group	10 (4/6)
Control group	16 (8/8)
*Inhibitory control tasks*	
Wait-for-treat task	39 (15/24)
Middle cup task	40 (15/25)
Leash task	36 (14/22)
*Problem-solving tasks*	
On-off task	40^a^ (16/24)
Size constancy task	36 (14/22)
Blocked tube task	28 (12/16)
Trap tube task	25^b^ (8/17)

^a^ Three of these dogs completed only three sessions and were therefore excluded from the analysis with the binary response variable (learning criterion reached yes/no).

^b^ Four of these dogs completed only three or four (instead of five) sessions of this task.

### Experience treatments

The subjects were split into three treatment groups, which were presented with different opportunities to gain experiences in the physical cognition domain. One group of dogs (the enriched group) was supplied with a set of twelve toys that gave them opportunities to learn about physical contingencies such as gravity, support or connectedness (cf. S1 Table). This set of toys also included three toys where attending to relative size differences was relevant for the subject to obtain a food reward. The second group of dogs (the manipulative group) was also supplied with a set of twelve toys, but these did not offer the same opportunities to learn about physical aspects that would become relevant in the physical problem-solving tasks (cf. S1 Table). That is, the toys were perceptually opaque and did not include toys for which relative sizes had any relevance. They gave the subjects the same manipulative experience of pushing/pulling various types of handles and objects as the toy set of the enriched group, however.

For both groups, the dog owners were supplied with the toys of the respective set for approximately one month each (median 5 weeks, range 3–10 weeks) when the dog was between 3 and 18 months of age (for the order of toys see S1 Table). The owners were asked to fill in log sheets to monitor duration and frequency of the dog’s interaction with each toy. At the end of the month, when one of the experimenters visited the dog and its owner, the final play session with the toy was videotaped to confirm that the dog had mastered it (cf. S1 Table for exceptions where this was not the case). The owner was then supplied with the next toy together with instructions and a log sheet, while the previous toy was collected and later provided to another dog in the same group.

A third group of dogs (the control group) was not supplied with any toys, but grew up with the customary balls, ropes and rubber toys for stimulation. An owner questionnaire was used to determine what kind of toys these dogs were familiar with at the age of 18 months. If the reported toys included more than one toy of the type used in our two toy sets, the dog was not included in the study, but the owner was encouraged to participate in a different study running in parallel [55].

The subjects of the enriched group and the manipulative group, but not the subjects of the control group, also participated in a string-pulling study [24] which offered them additional opportunities to learn about physical contingencies prior to their exposure to the physical problem-solving tasks described below.

### Inhibition tasks

The owners of all subjects were asked to practice three inhibitory control tasks with their dogs (see also S1, S2 and S3 Movies). This took place either before the start of the physical problem-solving tests (starting not before 8 months of age, N = 33 dogs), or parallel to the first sessions of the physical problem-solving tests (N = 8 dogs). The three tasks were chosen to represent a range of different situations that all require the ability to suppress the impulse to go straight for the reward, and do not have obvious confounds with other abilities such as object permanence, memory or numerical competence (though other confounds cannot be ruled out completely, see discussion).

The “wait-for-treat” task is a classic exercise in which a treat is placed on the ground in front of the dog and a “wait” command is given. The dog then has to wait for a “go” command before it is allowed to eat the reward. In case the dog tries to do so prematurely, the owner covers the treat with the hand or foot and removes it. The owners were instructed to gradually increase the waiting time and decrease the distance of the treat to the dog up to a duration of at least 5 s and a distance of no more than 10 cm from one of the dog’s front paws. We used this variant of a delay-of-gratification task, rather than the classic version with two different amounts of food (as for example in ref [56]), because in earlier experiments dogs did not consistently choose the plate with a larger number of treats presented next to a plate with a smaller number of treats on it [57,58].

The “middle cup task” is an inhibitory control task in which a treat is placed under two of three transparent cups that are lined up and turned upside down. The subject is then allowed to push over two of the three cups to obtain the rewards. Therefore, it has to refrain from pushing over the empty cup in order to obtain both rewards, which is particularly challenging if the empty cup is in the middle position of the array. Note that we used transparent rather than opaque cups for this task (unlike in [56]) to ensure that the performance was not confounded with object permanence abilities or memory effects. The owners were instructed to let the dog observe the baiting, to vary the position of the empty cup (middle, right, left) and to allow the dog to overturn only two of the three cups in each trial (i.e. blocking the third cup with their hand as soon as the dog had overturned the other two). For the “leash task”, the subjects were confronted with a situation in which the leash attached to their collar or harness would get caught on an obstacle (a tree, a lamp post or something similar). The owner would then call the dog from a position in front of the subject. In order to reach the owner and be rewarded, the dog therefore first had to move away from the owner and around the obstacle. The owners were instructed to gradually increase the distance the dog had to move back in order to be able to approach the owner (up to 2 m) and to show the dog the way around the obstacle in trials in which it did not succeed within 20 s.

With these three tasks, we aimed to assess the dog’s level of inhibitory control at the time of the physical problem-solving tests. For this purpose, we did not distinguish between acquired and innate level of inhibitory control (despite a substantial genetic component [59], and evidence for stability over periods over several years also in dogs [60], inhibitory control is also subject to environmental influences [59,61]; for example it can improve with practice of tasks involving self-control [62,63]). Differentiating between innate and acquired inhibitory control was not possible in this study as the “wait-for-treat” exercise and the “leash-task” are commonly done by many dog owners with their dogs and it was therefore not possible to observe initial performance for many of the subjects. Rather, we aimed to assess the performance in these tasks after a similar amount of practice in all of the subjects, which reduces the extent to which individual differences in performance may be reflecting differences in training history. Like for the toys, the owners were supplied with instructions and necessary materials and were asked to practice the three tasks for approximately one month each (and to fill in a log sheet). The final session of each inhibitory control task was conducted in the presence of an experimenter and was videotaped. Performance in the inhibitory control tasks was scored from these videos with a score of 0 corresponding to poor performance and a score of 2 to good performance in the respective task (for further details see S2 Table). A combined inhibition score was then calculated by adding the scores of the three tasks. In the eight cases in which a dog completed only two of the three tasks, the intermediate score of 1 was given for the missing task.

A second coder, who was naïve to the purpose of the experiment, coded the videos of a random subset of 18 subjects for each of the three inhibitory control tasks. Concordance was good for the scores of the wait-for-treat task (89%) and perfect for the scores of the middle cup task and the leash task (100%).

### Physical problem-solving tasks

Between the age of 18 and 28 months, the subjects were tested in four physical problem-solving tasks (size constancy task, on-off task, blocked tube task and trap tube task) with the order of tasks counterbalanced between individuals. The four tasks were chosen to represent a range of difficulty, but also as tasks that we expected were difficult to solve for dogs (thus offering room for improvement for the subjects in the enriched group). Previously published work had suggested that at least some dogs show an understanding of physical support [64] (cf. on-off task) and of the solidity principle [65] (cf. blocked tube task), and are sensitive to size differences of objects [66] (cf. size constancy task). The trap tube task to our knowledge has not been presented to dogs before and we expected it to be the most difficult of the four tasks. Not all subjects participated in all physical problem-solving tasks (cf. Table 1); therefore sample sizes differ for analyses of the different tasks.

#### Size constancy task

With the size constancy task we aimed to test whether the subjects understand that an object does not change its size when it is temporarily out of view. Rather than a violation-of-expectation paradigm (as in [66]), we used an active choice task, as this allowed us to repeat trials of different conditions for each subject. The subjects were first shown a football and then presented with two occluders, a tall one and a short one, with the ball hidden behind one of the two. While the short occluder left the top section of the ball visible (semi-occluded condition), the tall occluder covered the ball completely (occluded condition, see also S4 Movie). In the control condition, a screen was lowered to leave only the bottom part of the two occluders visible (giving no visual information about the location of the ball, see also S5 Movie). Each subject was presented with 12 trials per condition intermixed and in pseudorandomized order (for more details on material and procedures of the size constancy task see S1 File and S1 Fig).

If the subjects understand that the ball does not change its size, they should approach the tall occluder preferentially in the occluded condition, whereas they should choose the short occluder consistently in the semi-occluded condition. In the control condition, they should choose the side hiding the ball at chance level. Alternatively, some subjects may learn across trials to choose the tall occluder in the occluded condition, without showing evidence of an understanding of size constancy, which is what we found (for more detailed results of this task see S1 File and S2 Fig).

#### On-off task

The second physical problem-solving task was the support problem described in [67], which is commonly used to test for an understanding of causal relations involving physical support (e.g. [68–72]). Here, we used only the data of the on-off condition from that study (henceforth: on-off task). In this condition, the subjects were presented with an apparatus that consisted of two wooden boards that were mounted on rails on a platform (40 cm from each other) so that they could be moved by the dogs with their paws. The apparatus was placed inside a fenced area so that only the proximal ends of the boards were accessible to the subject. A piece of sausage was placed on one of the boards at its distal end and a second piece of sausage was placed 5 cm beside the second board. The subject was allowed to pull out only one of the two boards. The rewarded side was varied pseudorandomly between trials so that one side was rewarded never more than twice in a row. The subjects received a maximum of 60 trials of this task, in sessions of 10 trials that were separated by breaks of at least 5 min. No more than four sessions were conducted per day and test days were typically separated by one week (for further details see [67]). The interval between the first and the second test day of the on-off condition did not vary systematically between the three treatment groups (General Linear Model with square-root transformed intervals: F_2, 35_ = 0.85, p = 0.43) and also did not correlate significantly with the subjects’ inhibition score (F_1, 37_ = 1.11, p = 0.30). The learning criterion was set at a minimum of 16 correct choices in two consecutive sessions or a minimum of 22 correct trials in three consecutive sessions (binomial probability < 0.02). Unlike in the previous study [64], we found no evidence that dogs have an understanding of the causal structure underlying the task, but some subjects learned to solve the task, probably based on perceptual cues [67].

#### Blocked tube task

The third physical problem-solving task was the blocked tube task described in [73], which tests for an understanding of the solidity principle (that objects cannot pass through solid barriers; see also [74–76] for analogous tasks presented to primates). In this task, the subjects were offered a choice of two Plexiglas tubes that were mounted next to each other on a wall. The subjects could tilt the tubes by pushing or pulling down a handle attached the end of the tube, causing the tube’s content to roll out. A piece of sausage was placed in both tubes, but in one, an opaque barrier was inserted in front of the reward, preventing it from rolling out (in the other tube, an equal barrier was inserted behind the reward). As for the two physical problem-solving tasks described above, the rewarded side was varied pseudorandomly between trials so that one side was rewarded never more than twice in a row. Each subject received 50 trials of this task, in sessions of 10 trials that were separated by breaks of at least 5 min. No more than four sessions were conducted per day and test days were typically separated by one week (for further details see [73]).

#### Trap tube task

The fourth physical problem-solving task was a trap tube task as has been used in a series of physical cognition studies in primates [21,30,77–79] and later also in various bird species [22,80–83] to test for an understanding of causal relations involving gravity and solidity of objects. We adapted the primate setup for use in animals that do not use stick tools (and do not have a precision grip necessary to use them) such as dogs. To that end, the transparent trap tube was mounted on a stand and fitted with handles on both sides so that subjects could tilt the tube and make a reward placed in the centre of the tube roll out. As recommended [22,80,84], the tube was not only fitted with a functional trap on one side, but also with a perceptually equivalent non-functional trap on the other side. In order to obtain the reward, the subjects therefore had to choose which side of the tube to pull down, so that the reward rolls out of the tube, rather than rolling into the trap. The rewarded side was varied pseudorandomly between trials as for the blocked tube task. Each subject received 50 trials of this task (for more details on apparatus, procedures of the trap tube task see S2 File and S3 Fig, for an example trial see S6 Movie).

### Analyses

Statistical analyses were performed in R 3.1.1 [85]. Binomial generalized linear models (GLMs) were used to determine whether the performance in the physical problem-solving tasks was affected by the subjects’ experience and/or inhibitory control. These models included the treatment group (enriched, manipulative, control) and the inhibition score as predictors. The interaction term between the two predictors was included in initial models but found to be non-significant (p > 0.3) in all cases and subsequently dropped from the models. Likewise, the interaction terms between subject sex and the other two predictors were included in the initial models but found to be non-significant in all cases (p > 0.25) and subsequently dropped from the models. In cases where the inhibition score turned out to be a significant predictor of performance, separate models were calculated with the scores for the three different inhibition tasks as predictors to determine which of the three tasks provided the best predictive value for performance in the respective physical problem-solving task. In these cases, dogs that had not completed one of the inhibitory control tasks (see above) were excluded from the respective analysis.

Separate GLMs were calculated for three different response variables: 1) the proportion of correct choices in the occluded condition of the size constancy task, 2) the proportion of correct choices in the first two sessions of the on-off task (initial performance), and 3) a binary variable for whether the subject reached the learning criterion for the on-off task or not. In addition, as we found evidence for learning across the twelve trials of the occluded condition in the size constancy task (cf. S1 File), we explored whether experience and/or inhibitory control affected initial performance or learning in this task. For this purpose, the trials of the size constancy task were split into the first and the second half. We then used binomial generalized linear mixed models (GLMMs; [86]), with subject identity included as a random factor, to test for interactions between half (first vs. second half of trials) and the variables of interest (treatment group and inhibition score). Where applicable, the dataset was then split to test whether the variable of interest was a significant predictor for the first and/or for the second half of the size constancy trials. Performance in the blocked tube task and in the trap tube task were ultimately not included in these analyses due to minimal variation in performance between individuals (floor effects): No subject reached 60% correct choices in the last two sessions of the trap tube task (for more detailed results of the trap tube task, see S2 File and S4 Fig) and only one subject reached more than 60% correct choices in the last two sessions of the blocked tube task [73].

To determine whether performance in the on-off task was correlated with performance in the string-pulling study [24] previously conducted with the subjects of the enriched and manipulative groups, we ran further binomial GLMs for these subjects with performance in the 4-string task of the string-pulling study (the one task presented to all of the 24 subjects in that study) as a predictor. Three separate models were run to address this question: one with initial performance in the on-off task as the response variable and the proportion of correct choices in the first session of the 4-string task as predictor, one with the same response variable but with a binary predictor (reached the learning criterion in the 4-string task, yes/no), and finally one with the binary response variable (reached criterion for the on-off task yes/no) and the binary predictor mentioned above.

We applied a two-tailed alpha level of 0.05 throughout. Model outputs including also estimated coefficients and their standard errors are given in the Supporting Information (S3, S4 and S5 Tables).

## Results

### Effects of experience

**Fig 1:**
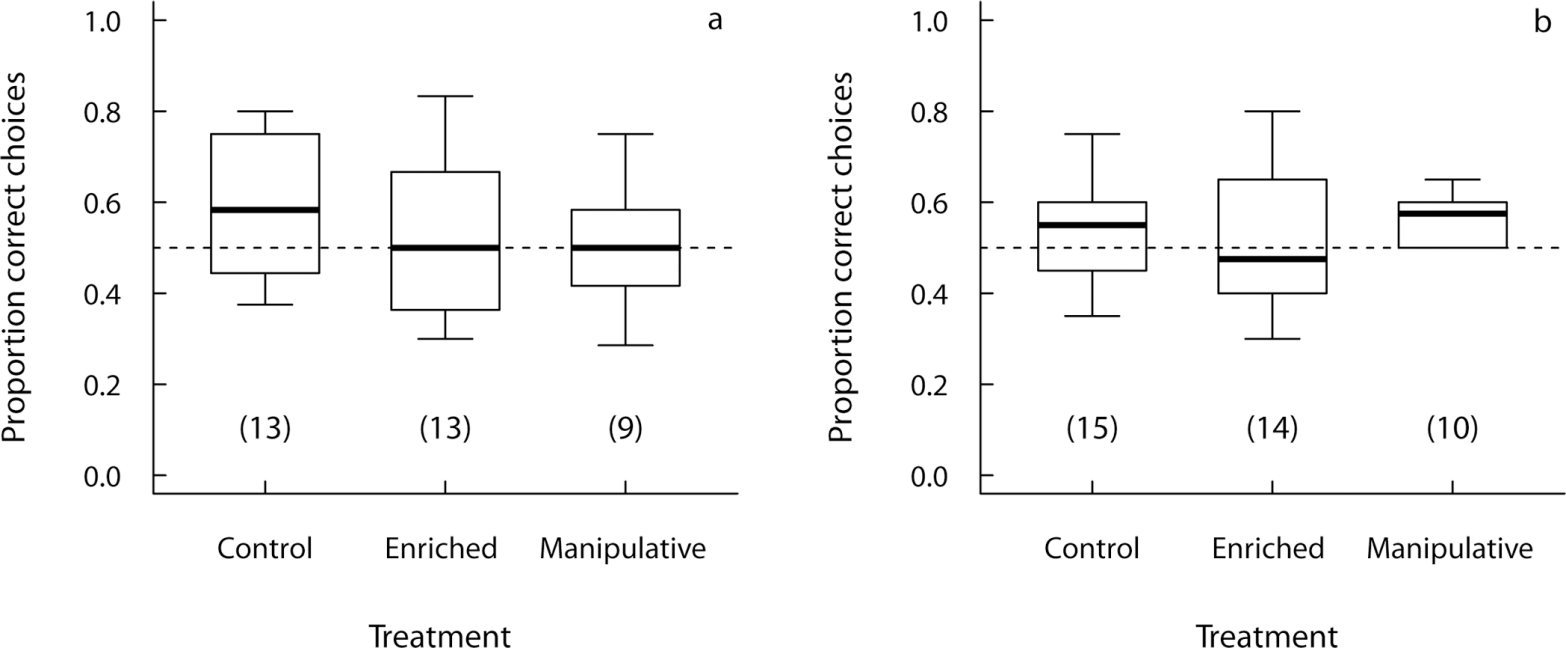
Physical problem-solving performance of the three treatment groups. Proportion of correct choices in **a** the occluded condition of the size constancy task and **b** the first two sessions of the on-off task. Boxplots indicate median, inter-quartile range, minimum and maximum.

Performance in the physical problem-solving tasks did not differ between the three treatments (enriched, manipulative, control; Fig 1). This was true for performance in the occluded condition of the size constancy task (GLM: χ^2^_(2)_ = 1.89, p = 0.39) as well as for the on-off task (initial performance: χ^2^_(2)_ = 0.09, p = 0.96; probability to reach the learning criterion: χ^2^_(2)_ = 0.20, p = 0.90). In addition, we also found no interaction between half (first vs. second half of trials) and treatment group on the performance in the size constancy task (GLMM; χ^2^_(2)_ = 2.43, p = 0.30). These results remained unchanged if subjects were excluded from the enriched group if they had not mastered the toys corresponding to the respective physical problem-solving task. In particular, in the case of the size constancy task, the result did not change if the four subjects that had not mastered toy 11 and/or toy 3 (cf. S1 Table) were excluded from the enriched group. As mentioned above, performance in the blocked tube task and in the trap tube task were ultimately not included in the analyses due to minimal variation in performance between individuals (floor effects).

Even though similar physical contingencies are underlying the two tasks, performance in the 4-string task was not correlated with performance in the on-off task: Initial performance in the on-off task was not correlated with initial performance in the string-pulling task (GLM: χ^2^_(1)_ = 0.01, p = 0.93) and was not significantly different for the subjects that had reached the learning criterion in the string-pulling task than for the subjects that had not reached the criterion (GLM: χ^2^_(1)_ = 0.08, p = 0.78). Furthermore, subjects that had reached the learning criterion in the string-pulling task were not more likely to reach the learning criterion in the on-off task than subjects that had failed in the string-pulling task (GLM: χ^2^_(1)_ = 1.42, p = 0.23).

### Effects of inhibitory control

**Fig 2:**
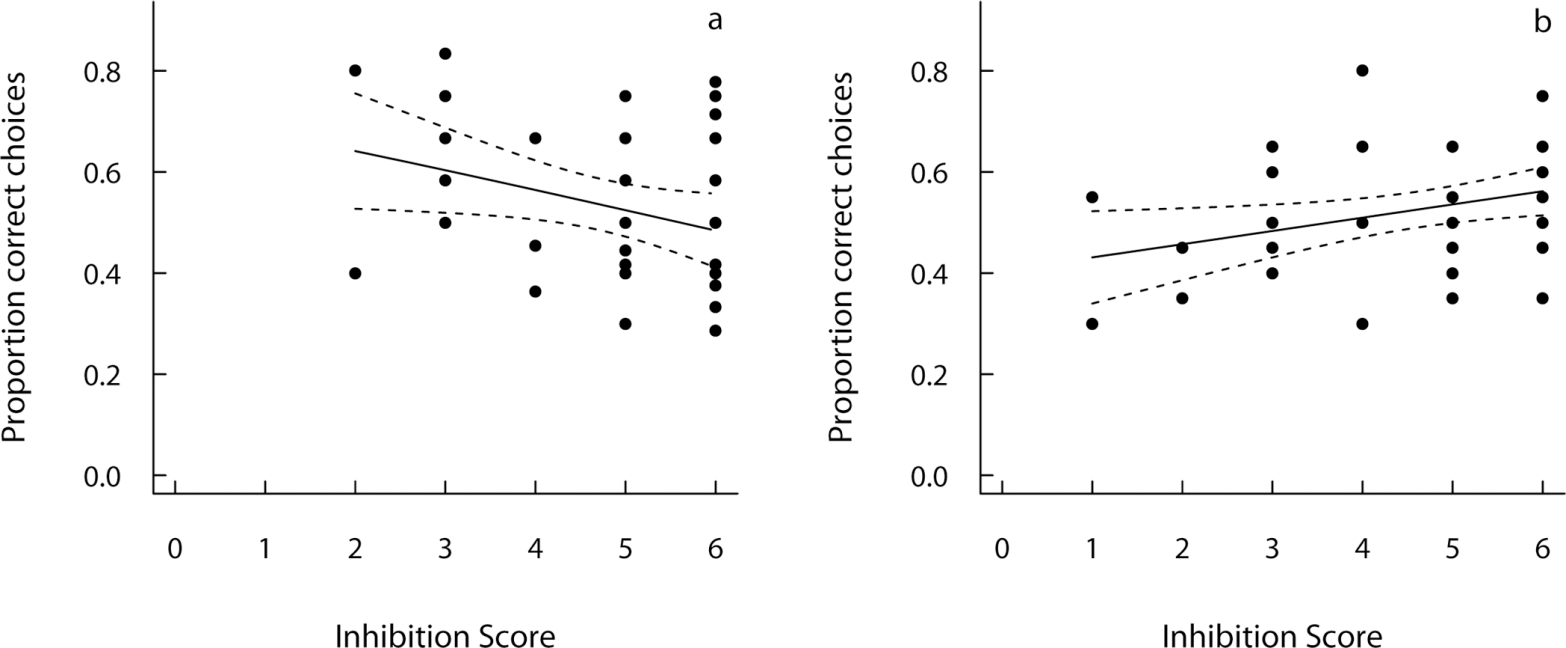
Physical problem-solving performance and inhibition scores. Proportion of correct choices in **a** the occluded condition of the size constancy task and **b** the first two sessions of the on-off task depending on the subject’s inhibition score. Shown are raw data and fitted curves with 95% confidence limits.

Inhibition scores ranged between 1 and 6 and did not differ systematically between the three treatment groups (Kruskal-Wallis test: χ^2^_(2)_ = 3.53, p = 0.17). The subjects’ inhibition score was a significant predictor of their performance in the size constancy task and the on-off task. The inhibition score was negatively correlated with performance in the occluded condition of the size constancy task (GLM: χ^2^_(1)_ = 4.00, p = 0.046; Fig 2A) and positively correlated with initial performance in the on-off task (GLM: χ^2^_(1)_ = 4.65, p = 0.03; Fig 2B). That is, subjects with poor inhibitory control performed poorly in the first sessions of the on-off task, but well in the size constancy task. Whether a subject reached the learning criterion for the on-off task, however, was not predicted by its inhibition score (GLM: χ^2^_(1)_ = 0.75, p = 0.39). The negative association between inhibition score and performance in the size constancy task cannot be explained by higher ball motivation of dogs with poor inhibition as the proportion of no-choice trials (the dog did not go searching for the ball when released, see also S1 File) did not predict performance in the occluded trials (binomial GLM: χ^2^_(1)_ = 0.08, p = 0.93) and was not correlated with the subjects’ inhibition score (binomial GLM with correction for overdispersion: F_1, 34_ = 0.06, p = 0.81).

As we found evidence for learning across the twelve trials of the occluded condition in the size constancy task (cf. S1 File), we further explored whether inhibition score predicted overall performance or learning in this task. We found evidence for the latter rather than for the former, reflected by a significant interaction between half (first vs. second half of trials) and inhibition score (GLMM; χ^2^_(1)_ = 3.86, p = 0.049). To characterize this interaction, we split the dataset into the first and the second half of the occluded trials and found that the inhibitory control score was a significant predictor for performance in the second half (GLM: χ^2^_(1)_ = 7.88, p = 0.005) but not for performance in the first half of the trials (GLM: χ^2^_(1)_ = 0.002, p = 0.97).

The scores for the three inhibitory control tasks correlated positively with each other. However, these correlations were rather weak (in one case even not significant, cf. Table 2). We therefore explored which of the three tasks predicted performance in the two physical problem-solving tasks best. Performance in the size constancy task was near-significantly related with the score for the middle cup task but not with the scores for the other two inhibition tasks (Table 3). In contrast, initial performance in the on-off task was significantly related with the score for the leash task, but not with the scores for two other two inhibition tasks (Table 3).

**Table 2:** Spearman correlation coefficients for scores of the three inhibitory control tasks.

Task	Wait-for-treat	Middle cup	Leash
Wait-for-treat	-	-	-
Middle cup	0.33*	-	-
Leash	0.45**	0.23	-

* p < 0.05

** p < 0.01.

**Table 3:** Relation between inhibitory control scores and performance in problem solving tasks (binomial GLMs).

Predictor	On-off task (initial performance)	Size constancy task (all trials)	Size constancy task (second half)
Combined score	χ^2^_(1)_ = 4.65, p = 0.03 (pos)*	χ^2^_(1)_ = 4.00, p = 0.046 (neg)*	χ^2^_(1)_ = 7.88, p = 0.005 (neg)**
Score of wait-for-treat task	χ^2^_(1)_ = 2.53, p = 0.11	χ^2^_(1)_ = 0.84, p = 0.36	χ^2^_(1)_ = 1.53, p = 0.22
Score of middle cup task	χ^2^_(1)_ = 0.09, p = 0.76	χ^2^_(1)_ = 3.46, p = 0.06 (neg)^†^	χ^2^_(1)_ = 8.45, p = 0.004 (neg)**
Score of leash task	χ^2^_(1)_ = 10.6, p = 0.001 (pos)**	χ^2^_(1)_ = 0.48, p = 0.49	χ^2^_(1)_ = 1.30, p = 0.26

neg/pos: negative/positive relationship between inhibitory control score and performance in the physical problem-solving task

^†^ p < 0.1

* p < 0.05

** p < 0.01.

## Discussion

The main aim of this study was to test whether experiences gathered over a prolonged period during manipulation of objects can lead to improved cognitive skills in the physical domain in animals in similar ways as they do in human infants. Our results do not support this hypothesis in the case of the domestic dog. While individual performance of our subjects varied considerably in two of the four physical problem-solving tasks, we found no differences in performance between the three treatment groups, even though the subjects had very different levels of experience with manipulative toys over a prolonged time period. Even experiences with conceptually very similar toys in the enriched group did not subsequently lead to improved performance in the physical problem-solving tasks. In addition, the subjects that had successfully solved a string-pulling task earlier [24] were not more likely to solve the conceptually similar on-off task. Taken together, these results suggest that dogs do not transfer knowledge about physical rules from one physical problem-solving task to another, but rather approach each task as a novel problem that they may (or may not) learn to solve over time. Our results also suggest that the reported poor performance of dogs in physical cognition tasks compared to primates [49–51] is only to a minor extent, or not at all, due to lacking experiences with manipulative toys (and hence fewer opportunities to learn about contingencies in the physical world).

While we found no evidence to support the prediction that experience with object manipulation leads to improved skills in the domain of physical cognition, we cannot rule out that experiences gathered closer in time to the tests (e.g. only few days or hours earlier) would influence the performance of dogs in physical problem-solving tasks. Also, while the toys offered to the enriched group gave opportunities to learn about physical contingencies (e.g. the concept of support or the relevance of size differences) that could have helped the subjects to solve the later physical problem-solving tasks, the setups of the toys and the later physical problem-solving tasks were quite different (for example in their spatial dimensions). It therefore remains possible that transfer between tasks would occur if the setups of the tasks resemble each other more closely and thus their similarities are recognizable to the subjects more easily.

It seems likely that our finding of a minimal or absent effect of prolonged experience on performance in physical problem-solving tasks in dogs, which conforms with the absence of experience effects in an earlier dog string-pulling study [29], extends to other species. Indeed, for animals that typically rely on perceptual cues to solve physical problem-solving tasks, as has been found for dogs (e.g. [67]) as well as for other mammals and birds (e.g. [22,72,80]), experience with perceptually similar tasks may even lead to worse performance in a subsequent task, if the previously learned associations do not lead to success in the latter. However some other non-human animals may also be able to profit from such experiences. Particularly likely candidates are primates, for which a number of studies have found indications for just that [30–32]. Investigating experience effects on performance in physical problem-solving tasks in other non-human animals will be necessary to determine whether the virtual absence of experience effects found in this study is the rule among non-human animals, and the experience effects found in some primates the exception, or vice versa. In addition, conducting studies similar to ours with primates can be useful to determine to what extent experience effects in non-human primates involve learning about physical concepts via playful object exploration, as is the case in human infants.

The second aim of this study was to test whether individual differences in problem-solving performance are linked to individual differences in inhibitory control. As predicted, the dogs’ performance in the physical problem-solving tasks was related to the individual level of inhibitory control. However, the effect was in the predicted direction only for the on-off task, where dogs with better inhibitory control performed better in the initial trials. In the size constancy task, in contrast, dogs with poorer inhibitory control scores performed better. Therefore, our results suggest that the relationship between individual differences in problem-solving performance and individual differences in level of inhibitory control is not straight-forward. We found not only that the direction of the relation differed between tasks, but also that the relation was apparent either only in the initial trials (on-off task) or only in the later trials (size constancy task), and that performance in the size constancy task and performance in the on-off task were best predicted by the scores of different inhibition tasks.

The rather complex interplay between inhibition scores and physical problem-solving performance in our study is likely due to the fact that inhibitory control is not a uniform but a polymorphic phenomenon. Studies in humans have shown not only domain-general variation in impulsivity [87–91], but also domain-specific variation between individuals [91–93]. For example, a recent study with 4th to 8th graders found that schoolwork-related impulsivity and interpersonal impulsivity are correlated with different personality traits and only moderately correlated with each other [93]. Similarly, a recent study on inhibitory control in dogs described domain-general aspects of inhibitory control [94], while other recent studies found that performance is only weakly (or not at all) correlated between different inhibition tasks in dogs [95,96], as we found in our study as well. These weak correlations between performances in different inhibition tasks suggest that the tasks may tap into different aspects of inhibitory control. Alternatively, for some of inhibitory tasks differences in performance may not primarily reflect the individual level of inhibitory control at all. For example, the wait-for-treat and the leash detour task in our study may reflect trainability (a frequently reported personality trait in domestic dogs [97,98]), as much or more than inhibitory control (that is, good trainability could have helped some subjects to reach high scores in these tasks), and the leash task also has a physical problem-solving component. Similarly, performance in tasks with transparent containers or barriers (widely used to assess inhibitory control, e.g. [40,52,78,79], this study) may be influenced by differences in visual perception (there is probably no task that purely measures inhibitory control and is not confounded with any other trait). Finally, as we tested for an association between the inhibitory control score and performance in problem-solving tasks with three different response variables, and the two significant cases cleared the conventional significance level only by a narrow margin, the possibility needs to be considered that one of them may be a false positive (it appears unlikely that both would be false positives).

To conclude, we found no evidence for an influence of manipulative experiences gathered over a long time period on the performance of dogs in physical problem-solving tasks. Instead, we found evidence that individual performance in such tasks is correlated with the subject’s level of inhibitory control. Further studies will be needed to elucidate the apparently complex relationship between inhibitory control scores and performance in problem-solving tasks, to determine under which conditions problem-solving performance is facilitated or hindered by good inhibitory control and which aspects of inhibitory control are influencing problem-solving performance under these conditions. In addition, complementary studies to the one presented here will be needed to determine whether the conspicuous absence of knowledge transfer between physical problem-solving tasks we found in our subjects is the rule or the exception for mammals.

## Supporting Information

S1 Fig Conditions of the size constancy task. (PDF)

S2 Fig Performance in size constancy task. (PDF)

S3 Fig Setup of the trap tube task. (PDF)

S4 Fig Performance in trap tube task. (PDF)

S1 File Size constancy task: methods and results. (PDF)

S2 File Trap tube task: methods and results. (PDF)

S3 File Datasets. (XLSX)

S1 Movie Example trial of wait-for-treat task. (AVI)

S2 Movie Example trial of middle cup task. (AVI)

S3 Movie Example trial of leash task. (AVI)

S4 Movie Example trial of size constancy task (occluded condition). (AVI)

S5 Movie Example trial of size constancy task (control condition). (AVI)

S6 Movie Example trial of trap tube task. (AVI)

S1 Table Toys supplied to subjects of the enriched and the manipulative group. (PDF)

S2 Table Scoring of inhibitory control tasks. (PDF)

S3 Table Models testing for effects of experience. (PDF)

S4 Table Models testing for effects of inhibitory control (inhibitory control score). (PDF)

S5 Table Models testing for effects of inhibitory control (scores of the three inhibition tasks separately). (PDF)
