# Supplementary material for: Inhibitory Control, but Not Prolonged Object-Related Experience Appears to Affect Physical Problem-Solving Performance of Pet Dogs
Source: PLoS One. 2016 Feb 10;11(2):e0147753. doi: 10.1371/journal.pone.0147753 (PMC4749342; doi:10.1371/journal.pone.0147753)
Supplement: S1 Fig — (PDF) [file pone.0147753.s001.pdf]

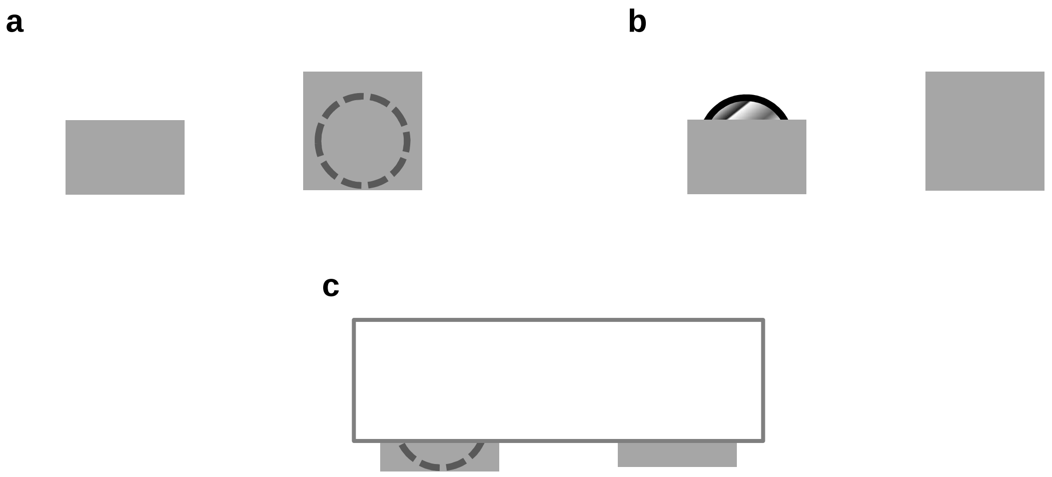

S1 Fig. Conditions of the size constancy task. a) occluded condition, **b**) semi-occluded condition, **c**) control condition.
