# Supplementary material for: Inhibitory Control, but Not Prolonged Object-Related Experience Appears to Affect Physical Problem-Solving Performance of Pet Dogs
Source: PLoS One. 2016 Feb 10;11(2):e0147753. doi: 10.1371/journal.pone.0147753 (PMC4749342; doi:10.1371/journal.pone.0147753)
Supplement: S2 Fig — (PDF) [file pone.0147753.s002.pdf]

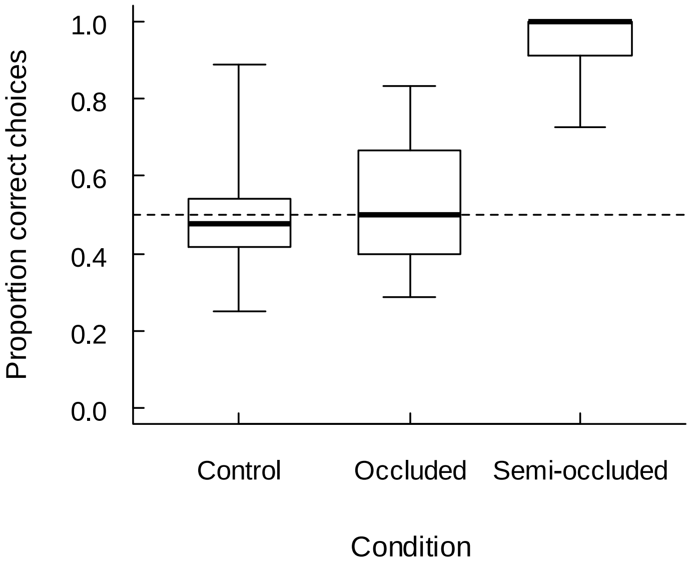


**S2 Fig. Proportion of correct choices for the three conditions in the size constancy task.** Boxplots indicate median, inter-quartile range and range. The dashed line indicates chance level.
