# Supplementary material for: Inhibitory Control, but Not Prolonged Object-Related Experience Appears to Affect Physical Problem-Solving Performance of Pet Dogs
Source: PLoS One. 2016 Feb 10;11(2):e0147753. doi: 10.1371/journal.pone.0147753 (PMC4749342; doi:10.1371/journal.pone.0147753)
Supplement: S3 Fig — (PDF) [file pone.0147753.s003.pdf]

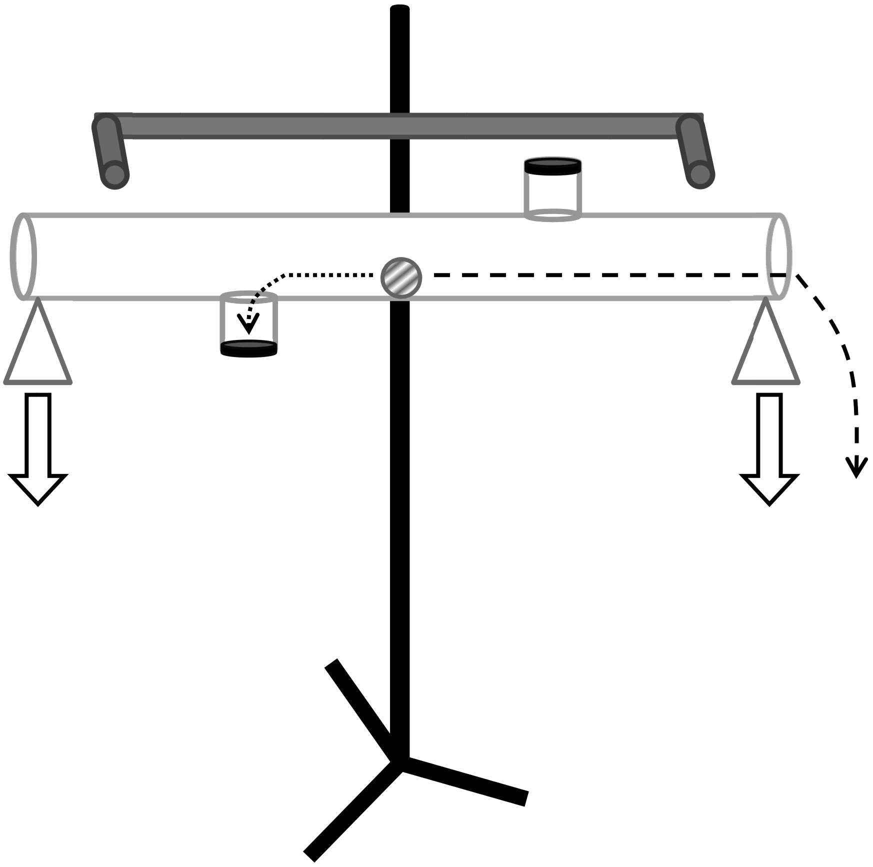

S3 Fig. Setup of the trap-tube task. If the dog pulls on the triangular handle at the correct end (here: right), the ball rolls out of the tube (dashed line); if the dog pulls on the handle at the incorrect end (here: left), the ball rolls into the trap (dotted line). The stoppers mounted above the trap tube (dark grey) prevent the tube from tilting by more than about 10 degrees, so that the ball cannot jump over the trap.
