# Supplementary material for: Inhibitory Control, but Not Prolonged Object-Related Experience Appears to Affect Physical Problem-Solving Performance of Pet Dogs
Source: PLoS One. 2016 Feb 10;11(2):e0147753. doi: 10.1371/journal.pone.0147753 (PMC4749342; doi:10.1371/journal.pone.0147753)
Supplement: S4 Fig — (PDF) [file pone.0147753.s004.pdf]

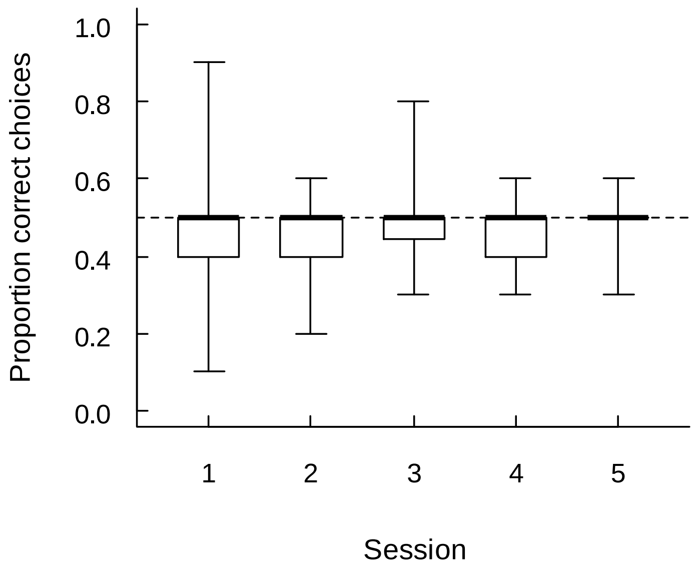

S4 Fig. Proportion of correct choices for the five sessions of the trap-tube task. Boxplots indicate median, inter-quartile range and range. The dashed line indicates chance level.
