# Supplementary material for: Inhibitory Control, but Not Prolonged Object-Related Experience Appears to Affect Physical Problem-Solving Performance of Pet Dogs
Source: PLoS One. 2016 Feb 10;11(2):e0147753. doi: 10.1371/journal.pone.0147753 (PMC4749342; doi:10.1371/journal.pone.0147753)
Supplement: S1 File — (PDF) [file pone.0147753.s005.pdf]

S1 File. Size constancy task: methods and results

Methods

For the size constancy task, a ball was hidden behind one of two occluders, one of which occluded the ball completely whereas the other one left the top part of the ball visible (cf. S1 Fig.). Both occluders were 55 cm wide, the tall occluder had a height of 35 cm, the short occluder had a height of 17 cm. The occluders were positioned at a distance of 60 cm from each other, 2 m in front of the subject (see also S4 Movie). As a target object, we used a football (diameter 22 cm) with reduced pressure to make it softer and thus more attractive to the dogs. Two replicas of the target object were used, one with nylon strings attached to it for the experiment and one without strings attached for the initial shaping procedure.

Before testing, all subjects were trained to search for the ball and touch it with the snout or paw using a shaping procedure. In this phase, the ball was hidden in different places in the room (but never behind one of the two occluders used in the subsequent experiment) and the dog was rewarded with a treat for approaching and touching it. In addition, the subjects were habituated to the moving screen used in the experiment (see below). Testing with a subject only started once it reliably searched for and touched the target object upon a verbal “search” command and once the subject showed no startling response to the moving screen anymore and walked underneath the suspended screen without hesitation. The owner remained outside the testing room for the pre-training phase as well as during the experiment, but could observe the proceedings on a computer screen.

In the experiment, the ball was hidden behind one of two occluders by an invisible experimenter using nylon strings attached to the ball, either behind the tall occluder (occluded condition), or behind the short occluder (semi-occluded condition). During the hiding process, a large screen (width 170 cm, height 70 cm) was lowered in front of the two occluders. The dog could therefore observe the ball disappearing behind the screen, but could not see behind which of the two occluders the ball ended up. After the hiding process was completed, the screen was raised again (cf. S4 Movie). In the control condition, the screen was raised by only 5 to 10 cm so that the dog had no visual information regarding the location of the ball (cf. S5 Movie, S1 Fig.). This condition served to

determine whether the subjects found the target object by following olfactory cues or by listening to (faint) acoustic cues audible during the hiding process. Each dog received three blocks of twelve trials, with four trials per condition in each block. The location of the tall and the short occluder was varied pseudorandomly so that the ball never ended up on the same side for more than two trials in a row. The order of conditions within blocks was varied randomly. No more than two blocks were conducted on a test day. Test days were separated by a median of 7 days (range 1-35, depending on availability of the dogs). The interval between the first and the last block did not vary systematically between the three treatment groups (General Linear Model with square-root transformed intervals: $F_{2, 32} = 1.63$, $p = 0.21$) and did not correlate significantly with the subjects' inhibition score ($F_{1, 34} = 3.90$, $p = 0.06$). Vice versa, the interval between the first and the last block did not predict performance in the occluded trials (binomial GLM: $\chi^2_{(1)} = 2.12$, $p = 0.15$).

For each trial, the handler entered the room with the dog once the experimenter had made the setup ready. At this point, the ball was placed in the middle between the two occluders and the screen was lowered so that the dog could not see either the ball or the occluders when it entered the room. The handler led the dog on its collar or harness to the start position, 2 m in front of the two hiding places, and put on a blindfold. The experimenter then started the trial by raising the screen to show the dog the ball and the two occluders. At this point, the dog had the opportunity to observe the size of the ball relative to the height of the two occluders. After lowering the screen again, the experimenter used the nylon strings attached to the ball to first raise it above the screen, lowering it vertically behind the screen again and then shifting it horizontally to one of the two hiding places. The experimenter then raised the screen and, after a delay of 5 seconds during which the subject could look at the setup, signalled the handler to release the dog by tapping his foot on the ground. If the dog did not leave immediately on its own accord, the handler additionally gave a verbal "search" command. Once the dog had found and touched the ball, the experimenter gave a verbal cue upon which the handler rewarded the dog with praise and a treat and led it out of the room. The experimenter then made the array ready for the next trial. The dog was always allowed to search until it had found and touched the ball. In cases where the dog approached the ball without touching it, the dog was not rewarded

immediately but was encouraged by the handler again until it touched the ball. This procedure was used to ensure that choices remained clear across trials.

The dogs' choices were coded live by the experimenter. A correct choice was coded when the dog approached the occluder behind which the ball was placed to within 10 cm without making a detour. An incorrect choice was coded when the dog approached the other occluder and turned to move to the correct occluder only after it could see the ball. Accurate coding of the latter was facilitated by markings on the floor extending from the two occluders towards the dog's start position (forming a large cross, once the dog's head had crossed a line the dog could potentially see the ball behind the opposite occluder, see also S4 Movie). "No choice" was coded if, upon release, the dog did not approach either of the two occluders, for example if it approached the hidden experimenter, walked to the door, or moved around in the room sniffing the floor. "Unclear choice" was coded if, upon release, the dog walked straight ahead and only turned towards one of the occluders once it could see the target object. If no choice and unclear choice trials were detected during live coding, they were repeated at the end of the test block, though for each dog a maximum of three trials per condition were repeated.

For data analysis, all trials were re-coded from video recordings of the experiments by CM. A second coder, who was naïve to the purpose of the experiment, coded one block of 12 trials for each of 18 subjects. Inter-observer concordance was good with an agreement of 89% for coding trials as correct choice, incorrect choice, unclear or no choice. For the majority of trials with disagreement (17 of 24), CM coded the trial as unclear or no choice, whereas the second coder coded them as correct choice (N = 8) or as incorrect choice (N = 9). Therefore, the coding of CM can be considered as conservative without introducing a bias towards correct or incorrect choices.

Of the 1421 trials performed in the size constancy experiment, 2 trials had to be excluded from the analyses due to experimenter error. No choice trials (N = 105) and unclear choice trials (N = 105) were also excluded. The proportion of correct choices was compared between conditions with a binomial Generalized Linear Mixed Model (GLMM) with a binomial error structure and dog identity included as a random factor to account for repeated measures. To test for learning across trials, the trials were split into the first half and the second half of trials for each condition and analysed with a

GLMM with condition, half, and the interaction term between the two as predictors and dog identity as a random factor. In addition, the proportion of correct choices was compared to chance level for each condition using a binomial Generalized Linear Model (GLM) with the intercept as the only predictor and testing for deviation of the intercept from 0 (corresponding to 50% correct choices). Finally, as we had found previously that female dogs performed better than males dogs in a size constancy test with a violation-of-expectation paradigm (Müller et al. 2011 Biol. Lett. 7: 689ff), we also tested for a sex difference in performance in the occluded condition.

Results

Performance differed significantly between conditions of the size constancy task (GLMM with likelihood ratio test: $\chi^2_{(2)} = 268.0$, $p < 0.0001$; S1 Fig.). However, performance in the occluded condition was significantly worse than in the semi-occluded condition ($z = -11.3$, $p < 0.0001$) and not significantly better than in the control condition ($z = 1.38$, $p = 0.17$). Furthermore, performance was significantly better than expected by chance only for the semi-occluded condition (GLM: $z = 13.2$, $p < 0.0001$) but not for the occluded condition (GLM: $z = 1.39$, $p = 0.17$) or the control condition (GLM: $z = -0.54$, $p = 0.59$). Performance in the occluded condition improved across trials though (GLMM with likelihood ratio test: $\chi^2_{(1)} = 4.49$, $p = 0.03$), and was significantly above chance level in the second half of the trials (GLM: $z = 2.53$, $p = 0.01$). Unlike predicted based on our previous results (Müller et al. 2011 Biol. Lett. 7: 689ff), females did not perform better in the occluded trials than males. On the contrary, in this active choice task, the males showed a (marginally significantly) better performance than the females (GLM: $z = 1.99$, $p = 0.047$).

Trials with unclear choices occurred significantly more often in the occluded than in the semi-occluded condition (GLMM with likelihood ratio test: $\chi^2_{(1)} = 34.0$, $p < 0.0001$; with our definition, unclear choices were not possible in the control condition). The occurrence of no choice trials, in contrast, did not differ significantly between the three conditions (GLMM with likelihood ratio test: $\chi^2_{(2)} = 5.13$, $p = 0.08$), though it was in tendency lower in the semi-occluded condition than in the other two conditions.
