# Supplementary material for: Inhibitory Control, but Not Prolonged Object-Related Experience Appears to Affect Physical Problem-Solving Performance of Pet Dogs
Source: PLoS One. 2016 Feb 10;11(2):e0147753. doi: 10.1371/journal.pone.0147753 (PMC4749342; doi:10.1371/journal.pone.0147753)
Supplement: S2 File — (PDF) [file pone.0147753.s006.pdf]

S2 File. Trap tube task: methods and results

Methods

The trap tube apparatus consisted of a 1 m Plexiglas tube (diameter: 11 cm) mounted horizontally on a stand and placed in the middle of the testing room (cf. S3 Fig. and S6 Movie). The tube was fitted with a functional trap (pointing downwards) 30 cm from one end and a non-functional trap (pointing upwards) at the same distance from the opposite end. The traps were also made of Plexiglas and had a 12 mm thick black wooden disc at the outer end. In addition, the tube had triangular string handles attached to both ends. Stoppers were mounted above the tube to ensure that it could be tilted by only about 10 cm. A rubber ball with 5 cm diameter, a hole drilled through its centre and a strip of sausage placed into this hole was placed in the centre of the tube as a reward object. A curtain was used to control visual access to the apparatus and wooden barriers were set up behind the curtain at both ends of the room for the experimenter to hide behind after baiting the apparatus (cf. S6 Movie).

Before testing started, all subjects were trained to pull at the string-handles with either the mouth or with a paw. For this purpose, an equal-sized Plexiglas tube without traps fitted was used and a string-handle was attached only to one end (varied pseudorandomly). During this phase, the height of the tube was adjusted to the size of the dog and its preferred mode of operation (with mouth or paw). The vertical position of the tube was then maintained throughout testing of the dog. The training phase was also used to habituate the subjects to the movement of the curtain. A subject proceeded to the test phase if it approached the handle directly, and pulled it down to tilt the tube and obtain the ball placed inside, in four consecutive trials (with the handle placed twice on each side).

For each test trial, experimenter 2 took the subject to the start position 2 m in front of the apparatus (while the curtain was still closed) and put on a blind-fold. Experimenter 1 then opened the curtain from the hidden location behind one of the wooden barriers. Experimenter 2 waited for 5 seconds after the curtain had been opened before releasing the dog with verbal encouragement to go "get it" (if experimenter 2 noticed that the dog was obviously not orienting towards the apparatus, the waiting period was extended until this was the case). If the dog pulled the correct handle and obtained the ball, experimenter 2 helped to get the piece of sausage out of it. If the dog pulled the incorrect

handle and the ball fell into the trap, experimenter 2 took the dog by the collar or harness and led it away from the apparatus.

During testing, the subjects were presented with the trap tube apparatus for 50 trials (5 sessions of 10 trials). The correct side (with the non-functional trap) was varied pseudo-randomly so that it was never on the same side more than twice in a row. As turning the tube and re-baiting it took between 1 and 2 minutes, experimenter 2 left the room with the dog after each test trial and entered again once the setup was ready for the next trial. Due to these breaks after each trial, no further breaks were introduced between sessions. Testing was spread over 2 or 3 test days (separated by 1 to 28 days; median: 7 days), with no more than 30 trials performed per test day.

The dogs' choices were noted live by experimenter 1 and these notes were used for analysis. A correct choice was noted if the dog pulled the correct handle and obtained the ball. An incorrect choice was coded if the dog pulled the incorrect handle and the ball fell into the trap. An incorrect choice was also coded if the dog first approached the correct handle, pulled it a little without displacing the ball and then switched to the incorrect side. "No choice" was coded if the dog did not approach either of the two handles within 5 seconds (N=29). No choice trials were generally repeated to obtain complete sets of 50 trials (this was erroneously not done for 3 of the 29 no choice trials). All test sessions were also video-recorded and reliability of the notes was confirmed by coding a random sample of 20 sessions from the videos (concordance between notes and video coding was 100%).

Seven trials (of the 1777 in total) were excluded from the analysis, either because the ball was displaced by the dog accidentally touching a part of the apparatus (not one of the handles, N = 5) or because the ball had moved away from the centre of the tube before the dog was released (N = 2). In six trials the ball jumped over the trap and came out of the tube after the dog had pulled at the incorrect handle. In these cases an incorrect choice was coded (the dog was not given the sausage from the ball in these trials). Group level performance was compared to chance level with binomial GLMs, as for the size constancy task.

Results

Even though the trap was clearly visible and salient to the subjects (several of them approached and inspected it directly in the first trial - as in S6 Movie), none of them solved the task within 5 sessions. No subject reached 60% correct choices over the complete 50 trials or over the last 20 trials. Similarly, no subject reached more than 70% correct choices over two consecutive sessions at any point during testing. At group level, mean performance varied between 45% correct choices (in the second session) and 49% correct choices (in the third session). When data were pooled across all sessions, performance was significantly below chance level (binomial GLM with correction for under-dispersion: $t_{24} = -2.17$, $p = 0.04$; see also S2 Fig.). This may be explained by some subjects being attracted to the functional trap without realizing its consequences (cf. S6 Movie - we did not observe any subject inspecting the non-functional trap, which was situated above the eye level of the dogs). In the later sessions, all but two of the 25 subjects fell into side bias (approaching one side of the apparatus first in at least 17 of 20 trials in two consecutive sessions, binomial probability < 0.003).
