# Supplementary material for: Inhibitory Control, but Not Prolonged Object-Related Experience Appears to Affect Physical Problem-Solving Performance of Pet Dogs
Source: PLoS One. 2016 Feb 10;11(2):e0147753. doi: 10.1371/journal.pone.0147753 (PMC4749342; doi:10.1371/journal.pone.0147753)
Supplement: S1 Table — (PDF) [file pone.0147753.s014.pdf]

S1 Table. Overview of the set of toys supplied to subjects of the enriched and the manipulative group*.

	Enriched group		Manipulative group	
1)	Dog Shift A[c] 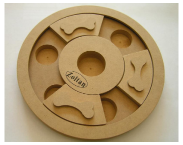	The dog can access treats by shifting the covers horizontally. As the covers move in a circle, one cover can be pushed on by the other.<br>*Mastered by 14/14 subjects*	Dog Shift B[c] 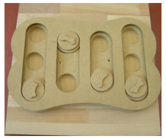	The dog can access treats by shifting the covers horizontally.<br>*Mastered by 10/10 subjects*
2)	Dog Watch A[c, 1] 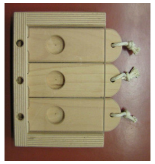	The dog can access treats placed underneath the transparent cover in any of the three slots by pulling out the corresponding drawer.<br>*Mastered by 14/14 subjects*	Dog Watch B[cm, 1] 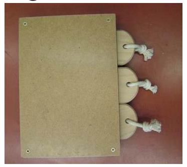	The same as Dog Watch A expect that the cover is opaque and the dog can therefore not see which of the three drawers is baited.<br>*Mastered by 10/10 subjects*
3)	Dog Smart A[m] 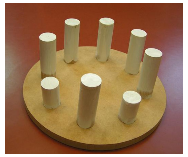	Only the rarer of the two cylinder types (large, small) is rewarded. The dog can access the treats by pulling out the cylinders.<br>*Mastered by 10/14 subjects*	Dog Smart B[m] 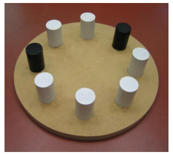	Only the rarer of the two cylinder types (black, white) is rewarded. The dog can access the treats by pulling out the cylinders.<br>*Mastered by 7/10 subjects*
4)	Gambling Tower[c, 2] 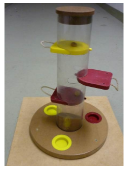	The dog can make the treats placed on any of the three levels of the transparent tower fall out at the bottom by pulling the levels out of the apparatus.<br>*Mastered by 14/14 subjects*	Dog Tower[m] 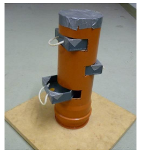	The dog can access treats by pulling out the three drawers.<br>*Mastered by 10/10 subjects*

5)	Dog Fall [c, 1]<br>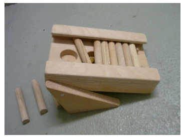	The dog can obtain treats by pushing the wooden rods upwards on the slanted apparatus. If not pushed up all the way, the rods keep rolling back down and cover the treats placed underneath.<br>*Mastered by 14/14 subjects*	Boomer [c, 1]<br>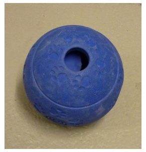	The dog can release treats placed in the ball by rolling it on the ground.<br>*Mastered by 10/10 subjects*
6)	Rod-pulling [m]<br>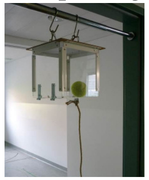	The dog can obtain a toy placed into the transparent box overhead by pulling at the rope.<br>*Mastered by 11/14 subjects*	Drawer [m]<br>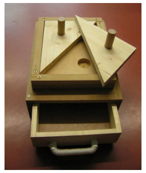	The dog can access treats by lifting the covers on top and another reward by pulling out the drawer.<br>*Mastered by 10/10 subjects*
7)	Dog Tube [m]<br>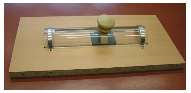	The dog can gain access to a treat placed inside the transparent half-tube by pushing the slider in the correct direction.<br>*Mastered by 10/14 subjects*	EL [c, 1]<br>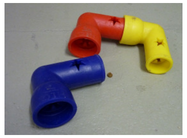	The dog can access treats place into the L-shaped rubber toys by turning and/or shaking them.<br>*Mastered by 10/10 subjects*
8)	Dog Box A [m]<br>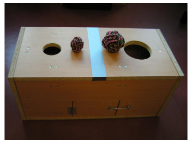	The dog is rewarded for putting the large ball into the large hole and the small ball into the small hole (the large ball does not fit into the small hole).<br>*None of the subjects mastered this task*	Dog Box B [m]<br>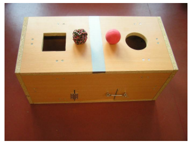	The dog is rewarded for putting the rubber ball into the round hole and the knotted ball into the square hole (arbitrary rule).<br>*None of the subjects mastered this task*
9)	Labyrinth [cm, 1]<br>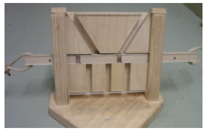	The dog can get treats fed into the labyrinth to fall out at the bottom by pulling the bar horizontally in the appropriate direction.<br>*Mastered by 14/14 subjects*	Folding toy [c, 3]<br>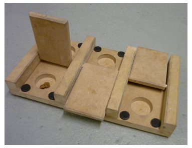	The dog can access treats placed underneath the covers by lifting them and/or folding them over.<br>*Mastered by 10/10 subjects*

10)	Tackle box [m] 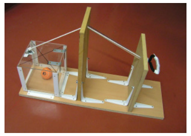	The dog can obtain the toy placed inside the transparent box (left) by pulling at the handle (right), which opens the box. *Mastered by 10/14 subjects*	Tornado [c, 1] 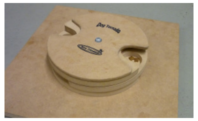	The dog can access the rewards hidden on several layers in the apparatus by rotating them. *Mastered by 10/10 subjects*
11)	Reversed dog box [m] 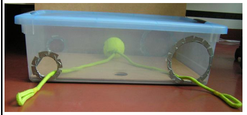	In order to obtain the ball placed inside the transparent box, the dog has to pull it out via the large hole, as it does not fit through the small hole. The apparatus has two sides so that the position of the large hole (left/right) can be varied. *Mastered by 12/14 subjects*	Dog Dizzy [c, 1] 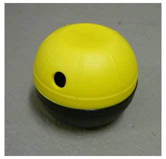	The dog can release treats placed in the ball by rolling it on the ground. The ball has two flattened sections (top and bottom) and weight attached to one half on the inside, which makes rolling it more difficult. *Mastered by 10/10 subjects*
12)	Trap table [m] 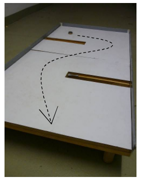	The table is fitted with a magnetic top sheet. The dog is rewarded for pushing the magnet on the table to the far end (avoiding the traps). *Mastered by 6/14 subjects*	Turn-pull toy [c, 1] 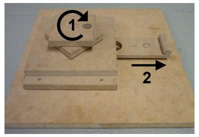	In order to obtain a treat placed inside, the dog has to first rotate the top part before pulling out the drawer at the bottom. *Mastered by 6/10 subjects*

* toys 1-4 were supplied to the subjects between 3 and 7 months of age, toys 5-9 between 8 and 13 months of age, and toys 10-12 between 14 and 16 months of age. The order of toys was chosen so that those that we expected to be easier to solve were presented in the first four months.

[c] commercial dog toy, [cm] modified commercial dog toy, [m] custom-made dog toy

[1] obtained from www.hundespiele.com; [2] obtained from www.marthas-tierwelt.at; [3] obtained from www.dog-intelligenz.de
