# Supplementary material for: Inhibitory Control, but Not Prolonged Object-Related Experience Appears to Affect Physical Problem-Solving Performance of Pet Dogs
Source: PLoS One. 2016 Feb 10;11(2):e0147753. doi: 10.1371/journal.pone.0147753 (PMC4749342; doi:10.1371/journal.pone.0147753)
Supplement: S2 Table — (PDF) [file pone.0147753.s015.pdf]

S2 Table. Scoring of inhibitory control tasks in videos of final sessions.

"Wait-for-treat"	Score 2	Waited for "go" command after at least 5 s with treat placed within 10 cm of one of the front paws in the first two trials OR in at least three of four consecutive trials.
	Score 1	Waited for at least 5 s in one or two of four trials
	Score 0	Never waited for at least 5 s
"Middle cup"[1]	Score 2	Successful in four consecutive trials (at least two of them with the middle cup left empty) OR no more than one mistake in six consecutive trials
	Score 1	More than 1 mistake in 6 consecutive trials but with a least one success when the middle cup was left unbaited
	Score 0	Never successful when the middle cup was left unbaited
"Leash"[2]	Score 2	At least one success for distance 2 m OR at least two successes for distance 1 m with no more than one failure.
	Score 1	At least one success for distance 1 m and more than one failure.
	Score 0	No successes

[1] Success: overturned the two baited cups first; mistake: overturned the unbaited cup before overturning one or both of the baited cups.

[2] Success: walked back and around the obstacle to reach the owner within 20 s; failure: failed to reach the owner within 20 s. Distance refers to the distance the dog had to move away from the owner in order to be able to reach him/her. If more than three trials were performed, only the first three were considered.
