# Supplementary material for: Inhibitory Control, but Not Prolonged Object-Related Experience Appears to Affect Physical Problem-Solving Performance of Pet Dogs
Source: PLoS One. 2016 Feb 10;11(2):e0147753. doi: 10.1371/journal.pone.0147753 (PMC4749342; doi:10.1371/journal.pone.0147753)
Supplement: S3 Table — (PDF) [file pone.0147753.s016.pdf]

S3 Table. Models testing for experience effects

Response variable	Predictor	Test statistic	p value	coefficient	standard error
On-off (criterion reached Y/N)	Treatment group	$\chi^2_{(2)} = 0.20$	0.90		
Treatment: Enriched				0.29	0.81
Treatment: Manipulative				-0.08	0.88
On-off (initial performance)	Treatment group	$\chi^2_{(2)} = 0.09$	0.96		
Treatment: Enriched				0.002	0.17
Treatment: Manipulative				-0.05	0.19
Size constancy (all trials)	Treatment group	$\chi^2_{(2)} = 1.89$	0.39		
Treatment: Enriched				-0.32	0.25
Treatment: Manipulative				-0.28	0.27
Size constancy (all trials) (GLMM)	Treatment group * Half of trials	$\chi^2_{(2)} = 2.43$	0.30		
Treatment: Enriched * Half: Second				-0.18	0.49
Treatment: Manipulative * Half: Second				0.67	0.57
On-off (initial performance)	String-pulling (initial performance)	$\chi^2_{(1)} = 0.01$	0.93	0.01	0.08
On-off (initial performance)	String-pulling (criterion reached Y/N)	$\chi^2_{(1)} = 0.08$	0.78	0.08	0.27
On-off (criterion reached Y/N)	String-pulling (criterion reached Y/N)	$\chi^2_{(1)} = 1.42$	0.23	1.10	0.96
