# Supplementary material for: Inhibitory Control, but Not Prolonged Object-Related Experience Appears to Affect Physical Problem-Solving Performance of Pet Dogs
Source: PLoS One. 2016 Feb 10;11(2):e0147753. doi: 10.1371/journal.pone.0147753 (PMC4749342; doi:10.1371/journal.pone.0147753)
Supplement: S4 Table — (PDF) [file pone.0147753.s017.pdf]

S4 Table. Models testing for effects of inhibitory control (inhibition score)

Response variable	Predictor	Test statistic	p value	coefficient	standard error
On-off (criterion reached Y/N)	Inhibition score	$\chi^2_{(1)} = 0.75$	0.39	0.21	0.25
On-off (initial performance)	Inhibition score	$\chi^2_{(1)} = 4.65$	0.03	0.11	0.05
Size constancy (all trials)	Inhibition score	$\chi^2_{(1)} = 4.00$	0.046	-0.17	0.09
Size constancy (all trials) (GLMM)	Inhibition score * Half of trials	$\chi^2_{(1)} = 3.86$	0.049	-0.34	0.17
Size constancy: first half of trials	Inhibition score	$\chi^2_{(1)} = 0.002$	0.97	-0.005	0.12
Size constancy: second half of trials	Inhibition score	$\chi^2_{(1)} = 7.88$	0.005	-0.34	0.13
