# Supplementary material for: Inhibitory Control, but Not Prolonged Object-Related Experience Appears to Affect Physical Problem-Solving Performance of Pet Dogs
Source: PLoS One. 2016 Feb 10;11(2):e0147753. doi: 10.1371/journal.pone.0147753 (PMC4749342; doi:10.1371/journal.pone.0147753)
Supplement: S5 Table — (PDF) [file pone.0147753.s018.pdf]

S5 Table. Models testing for effects of inhibitory control (scores of the three inhibition tasks separately)

Response variable	Predictor	Test statistic	p value	coefficient	standard error
On-off (initial performance)	Score of wait-for-treat task	$\chi^2_{(1)} = 2.53$	0.11	0.25	0.16
On-off (initial performance)	Score of middle cup task	$\chi^2_{(1)} = 0.09$	0.76	0.03	0.08
On-off (initial performance)	Score of leash task	$\chi^2_{(1)} = 10.6$	0.001	0.36	0.11
Size constancy (all trials)	Score of wait-for-treat task	$\chi^2_{(1)} = 0.84$	0.36	-0.31	0.34
Size constancy (all trials)	Score of middle cup task	$\chi^2_{(1)} = 3.46$	0.06	-0.23	0.13
Size constancy (all trials)	Score of leash task	$\chi^2_{(1)} = 0.48$	0.49	-0.11	0.17
Size constancy: second half of trials	Score of wait-for-treat task	$\chi^2_{(1)} = 1.53$	0.22	-0.61	0.51
Size constancy: second half of trials	Score of middle cup task	$\chi^2_{(1)} = 8.45$	0.004	-0.55	0.20
Size constancy: second half of trials	Score of leash task	$\chi^2_{(1)} = 1.30$	0.25	-0.26	0.24

These models were only calculated for response variables where the overall inhibition score appeared as a significant predictor of performance (cf. S4 Table).
